# Laparoscopic versus open hemihepatectomy: does side matter? A post-hoc analysis of the ORANGE II PLUS randomized controlled trial

**DOI:** 10.1007/s00464-026-12588-w

**Published:** 2026-03-09

**Authors:** Bram Olij, Gabriela Pilz da Cunha, Francesca Ratti, Mohammad Abu Hilal, Roberto I. Troisi, Robert P. Sutcliffe, Marc G. Besselink, Somaiah Aroori, Krishna V. Menon, Bjørn Edwin, Mathieu D’Hondt, Valerio Lucidi, Tom F. Ulmer, Rafael Díaz-Nieto, Zahir Soonawalla, Steve White, Gregory Sergeant, Christoph Kuemmerli, Remon Korenblik, Vincenzo Scuderi, Frederik Berrevoet, Aude Vanlander, Ravi Marudanayagam, Pieter J. Tanis, Marielle M. E. Coolsen, Robert S. Fichtinger, Zina B. Eminton, Ulf P. Neumann, Lloyd Brandts, Siân A. Pugh, Åsmund A. Fretland, John N. Primrose, Ronald M. van Dam

**Affiliations:** 1 Department of Surgery and Transplantation, Maastricht University Medical Centre+, PO Box 5800, 6202 AZ Maastricht, The Netherlands; 2 GROW – School for Oncology and Developmental Biology, Maastricht University, Maastricht, the Netherlands; 3 Department of Surgery, Amsterdam UMC, Location University of Amsterdam, Amsterdam, The Netherlands; 4 Cancer Centre Amsterdam, Amsterdam, The Netherlands; 5 Hepatobiliary Surgery Division, IRCCS San Raffaele Hospital, Milan, Italy; 6 Department of Surgery, School of Medicine, The University of Jordan, Amman, 11942 Jordan; 7 University Surgery and Perioperative and Critical Care Theme, NIHR Southampton Biomedical Research Centre, University Hospital Southampton/University of Southampton, Southampton, UK; 8 Division of HPB, Minimally Invasive and Robotic Surgery, Department of Clinical Medicine and Surgery, Transplantation Service, Federico II University, Naples, Italy; 9 HPB and Liver Transplant Unit, University Hospitals Birmingham NHS Trust, Birmingham, UK; 10 Department of Surgery, Plymouth Hospitals NHS Trust, Plymouth, UK; 11 Department of Liver Transplant and HPB Surgery, Institute of Liver Studies, King’s College Hospital NHS Foundation Trust, London, UK; 12 Intervention Centre and Department of Hepatic, Pancreatic and Biliary Surgery, Oslo University Hospital and Institute of Clinical Medicine, University Hospital of Oslo, Oslo, Norway; 13 Department of Digestive and Hepatobiliary/Pancreatic Surgery, AZ Groeninge, Kortrijk, Belgium; 14 Department of Digestive Surgery, Unit of Hepatobiliary Surgery and Transplantation, Hôpitaux Universitaires de Bruxelles, Hôpital Erasme, Brussels, Belgium; 15 Department of Surgery and Transplantation, University Hospital RWTH Aachen, Aachen, Germany; 16 Department of Surgery and Transplantation, University Hospital Essen, Essen, Germany; 17 Department of Hepato-Biliary Surgery, Aintree University Hospital NHS Foundation Trust, Liverpool, UK; 18 Department of Surgery, Oxford University Hospitals NHS Foundation Trust, Oxford, UK; 19 Department of Surgery, Newcastle Upon Tyne Hospitals NHS Foundation Trust, Newcastle Upon Tyne, UK; 20 Department of Abdominal Surgery, Jessa Hospital, Hasselt, Belgium; 21 Faculty of Medicine and Life Sciences, UHasselt, Hasselt, Belgium; 22 Department of General, HPB and Liver Transplantation Surgery, Ghent University Hospital, Ghent, Belgium; 23 Department of Surgery, Free University Hospital, AZ Jette Hospital, Brussels, Belgium; 24 Southampton Clinical Trials Unit, University of Southampton, Southampton, UK; 25 Department of Clinical Epidemiology and Medical Technology Assessment, Maastricht University Medical Centre+, Maastricht, the Netherlands; 26 Department of Oncology, Addenbrooke’s Hospital, Cambridge, UK; 27 Department of General Surgery, Santa Maria delle Grazie Hospital, ASL Napoli2 Nord, Napoli, Italy

**Keywords:** Right hemihepatectomy, Left hemihepatectomy, Laparoscopic hepatectomy, Randomized controlled trial, Minimally invasive liver surgery

## Abstract

**Background:**

Laparoscopic liver surgery offers several benefits, yet the adoption of laparoscopic right hemihepatectomy (RH) is slow, owing to its high degree of technical complexity. It is uncertain whether the general benefits of laparoscopy also extend to RH. This study evaluates perioperative outcomes of laparoscopic vs open RH, and illustrates differences in laparoscopic RH and left hemihepatectomy (LH) within the international, multicentre, double-blinded ORANGE-II-PLUS randomized trial.

**Methods:**

Patients were randomly assigned to open (*n* = 166) or laparoscopic hemihepatectomy (*n* = 166). The present *post-hoc* subgroup analysis compares perioperative and oncological outcomes of laparoscopic RH (*n* = 105) vs open RH (*n* = 108). In addition, interaction between surgical approach (open or laparoscopic) and hemihepatectomy laterality (RH; *n* = 213 vs LH; *n* = 119) was assessed.

**Results:**

There was a higher proportion of malignancy, including more colorectal liver metastases, and more preoperative portal vein embolization in patients undergoing RH compared to LH, other characteristics were well-balanced. The laparoscopic approach was associated with shorter time to functional recovery compared to open surgery for RH (median 5 vs 5 days, *p* = .004) and shorter length of hospital stay (median 5 vs 6 days, *p* = .014). Except for longer operating times in laparoscopy (332 vs 263 min, *p* < .001), no differences were found in other perioperative and oncological outcomes between laparoscopic and open RH. For all outcomes, interaction testing between surgical approach and laterality did not reach significance, suggesting that approach did not affect RH and LH differently. Though patients requiring laparoscopic RH needed longer operating time (332 vs 225 min) and time to functional recovery (median 5 vs 3 days) than patients requiring laparoscopic LH.

**Conclusion:**

Patients undergoing RH showed modest, population-level, benefits from a laparoscopic approach with regard to time to functional recovery and hospital length of stay, despite higher technical complexity and a more pronounced postoperative impact on the patient. Interaction testing between RH and LH did not reach significance, suggesting the effect of the approach on outcomes were consistent regardless of resection laterality. These results support the implementation of the laparoscopic approach for RH if surgeons are experienced.

**Clinical trial information:**

NCT01441856.

**Graphical abstract:**

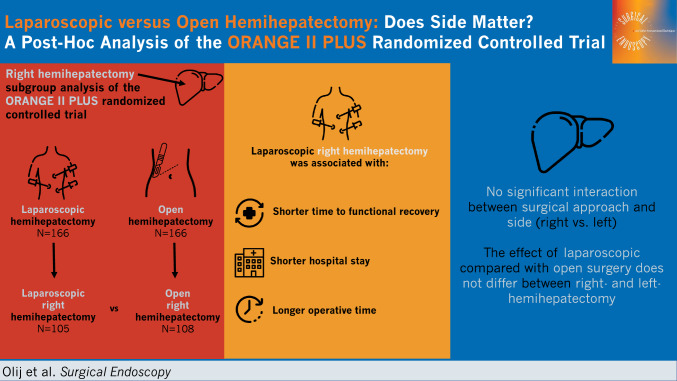

**Supplementary Information:**

The online version contains supplementary material available at 10.1007/s00464-026-12588-w.

The physical impact of liver resection for patients with primary and metastatic tumours of the liver and biliary tract has reduced over time. Minimally invasive liver surgery (MILS), including laparoscopic hemihepatectomy, promotes accelerated functional recovery and consequently shortens length of hospital stay without jeopardizing safety or oncological outcomes [1–8]. However, it demands specialized training and expertise due to the specific technical skills required for its execution [9, 10].

Right hemihepatectomy (RH) is considered one of the most demanding liver resections [10]. This is primarily due to challenges posed by the anatomy of the right hemi-liver, including its posterosuperior location within the abdominal cavity, relation to the hilar structures, the large transection plane and large liver volume [11]. The difficulty is amplified in laparoscopic liver resection owing to the limited range of motion and haptics of laparoscopic instrumentation. This is reflected in various difficulty scoring systems for laparoscopic liver surgery, where higher scores are attributed to resections of the right hemi-liver [11, 12]. In the 2018 Southampton Consensus Guidelines for Laparoscopic Liver Surgery, right and left major hepatectomy are described as sufficiently distinct that they require separate assessment [13]. However, there are no randomized studies assessing the impact of laparoscopy on outcomes of RH specifically, as well as limited literature comparing RH with left hemihepatectomy (LH) [14].

Retrospective studies have shown that the clinical benefits of laparoscopy are evident in right major liver resections [15, 16]. A multicentre propensity score matched analysis showed a reduction of biliary fistulas for laparoscopic compared to open surgery for both LH and RH and a reduction in the occurrence of ascites specifically in RH when employing the laparoscopic approach [17]. Laparoscopic RH is, however, associated with relatively high conversion rates (6 to 18%), underscoring its technical complexity [18–20]. Douaiher et al. and Karanjia et al. both found higher rates of morbidity and mortality following right hepatectomy [21, 22]. In another study, RH was identified as a risk factor for postoperative bile leak [23]. These studies did not investigate the effect of surgical approach (open or laparoscopic) on postoperative and oncological outcomes.

The ORANGE II PLUS randomized trial concluded that laparoscopic hemihepatectomy is associated with quicker recovery, shorter hospital stay, and improved health-related quality of life compared to open hemihepatectomy, however, separate analysis depending on operation side – RH or LH – might provide additional insights. A nuanced interpretation is needed to better understand the impact of laparoscopy in the context of RH specifically, rather than hemihepatectomy as an entirety [5, 24]. This study investigates the impact of surgical approach on patients undergoing RH and reports outcomes of RH and LH separately.

## Methods

### Study design and participants

The ORANGE II PLUS double-blind, randomised controlled phase 3 trial was conducted in 16 centres across 6 European countries, specialised in hepatobiliary oncology [5]. Eligible patients were adults who required RH or LH (with or without the need for one additional hepatic wedge resection or metastasectomy) for accepted indications. Furthermore, only patients with a Body Mass Index (BMI) between 18 and 35 kg/m^2^, an American Society of Anesthesiologists (ASA) physical status of I, II or III, and if they were able to understand the requirements of the trial, were included. Patients who underwent repeat hepatectomy or were not eligible for a laparoscopic approach due to insufficient margin from vascular or biliary structures were excluded from the trial. Pregnant or breastfeeding women were also excluded. Centres were selected based on substantial experience with minimally invasive minor liver resections and at least intermediate experience with major liver resections, defined as having performed a minimum of 10 laparoscopic hemihepatectomies. Six centres had individual surgeons who had completed more than 40 laparoscopic major liver resections, and the remaining ten centres had surgeons who had performed more than 10 procedures before initiation of the trial.

### Ethical considerations

Ethical approval of the study protocol was obtained from Maastricht University Medical Centre Medical Ethical Committee (METC) (NL36215.068.11). The study was registered at ClinicalTrials.gov (NCT01441856). All patients in this trial provided written informed consent. All patients were given a detailed description of the study at least 1 week prior to inclusion. Anonymity and confidentiality were guaranteed for the patients regarding the obtained information. The trial was conducted in accordance with the Declaration of Helsinki and with Good Clinical Practice as defined by the International Conference of Harmonization.

### Randomisation and blinding

Patients were randomly assigned in a 1:1 ratio to either open or laparoscopic hemihepatectomy using a minimization scheme with hemihepatectomy side and treatment centre to balance treatment arms.

Blinding for treatment allocation was applied. Patients and ward personnel were unaware of the allocated treatment until day four after surgery using a large abdominal dressing covering all surgical incisions.

### Data collection, definitions, and outcome

Data were prospectively registered by the local investigators. Preoperatively, patient characteristics and baseline questionnaire answers were registered. Intraoperatively, surgical and anaesthesiologic information was obtained. During hospitalization, components related to functional recovery were documented daily. Follow-up questionnaires were conducted at ten days, three months, six months, and twelve months postoperatively.

The primary outcome, time to functional recovery, is defined as the time in days between the end of surgery and the moment a patient is deemed sufficiently recovered for discharge. Functional recovery is a composite endpoint and is reached once a patient meets a set of five criteria. These include adequate pain control with only oral analgesics, independent mobility as ascertained by a mobility score of 8 or higher or at the preoperative level, solid food tolerance for more than 24 h, normalized or improving serum tests, and independence from intravenous fluid administration. The serum tests include total bilirubin, alanine aminotransferase, aspartate aminotransferase and international normalized ratio.

The secondary outcomes include perioperative and oncological outcomes. These comprise of intraoperative blood loss, operating time, intraoperative incidents, conversion rate from laparoscopic to open surgery, length of hospital stay, in-hospital and 90-day mortality, 90-day (liver specific) morbidity and rate of readmission. Postoperative complications were divided into minor (Clavien-Dindo grade 1 and 2), major (Clavien-Dindo grade ≥ 3a), and cumulative according to the Comprehensive Complication Index (CCI) score [25, 26]. In addition, resection margin status, disease-free survival, and overall survival were included for patients with cancer. Disease-free survival is defined as the time from surgery to death from any cause or recurrence of cancer, whichever occurred first. Liver specific morbidity is defined as the occurrence of one or more of the following complications: intra-operative mortality, intra-abdominal haemorrhage, ascites, bile leakage, intra-abdominal abscess, or postoperative liver failure [27].

### Surgical technique

Surgical technique was not standardized, surgeons had the liberty to operate according to their own preferred methods. All patients were treated within an enhanced recovery after surgery (ERAS®) programme [28].

### Statistical analysis

The modified intention-to-treat analysis included all randomized patients, according to the treatment to which they had been allocated, but patients who did not undergo any surgery were excluded from the analysis after randomization. Patients who had not received the intended treatment, but who had undergone another type of liver surgery were included. However, patients were excluded if they had withdrawn their consent or if they had not received any surgery.

The primary outcome time to functional recovery was analysed with linear regression per treatment arm, adjusting first for centre (dummy coded) and then for age (years, continuous), sex (male/female) and tumour type (benign/malignant). Considering the non-normality (positive skewness) of the residuals, the outcome was log-transformed and the regression was repeated. The effect estimates and their confidence intervals were back-transformed to express them as a relative (i.e., %) outcome differences between laparoscopic and open surgery. Additionally, non-parametric tests were performed to check for robustness against non-normality. A *p*-value of < 0.05 was considered as statistically significant.

The secondary surgical and oncological endpoints were likewise assessed with fixed regression, linear for continuous outcomes, logistic for binary outcomes, and Cox for time-to-event outcomes.

Operative, postoperative and oncological outcomes of laparoscopic and open RH were compared. Additionally, P-interaction testing was used to evaluate differences between RH and LH in both the laparoscopic and open technique. To visualize these differences, the outcomes of RH and LH are presented separately in the appendix. The data are descriptive only, therefore, no analyses were performed.

All analyses were performed using IBM SPSS Statistics for Windows version 27.0.1.0, and R statistical computing for Windows version 4.1.0.

## Results

### Demographics

**Fig. 1:**
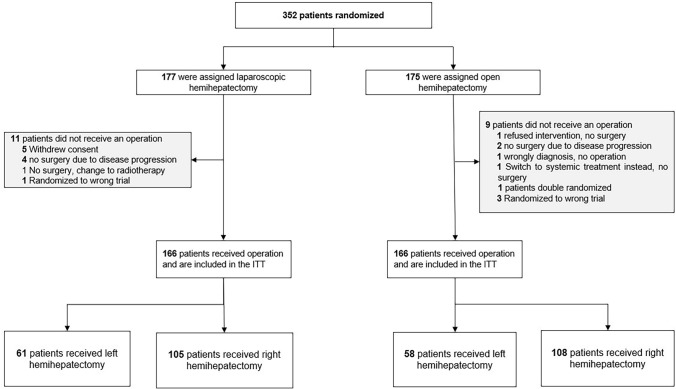
CONSORT flowchart

From October 2013 through January 2019, a total of 352 patients were randomized (Fig. 1).

Three hundred and thirty two patients underwent hemihepatectomy (213 right and 119 left).

### Right hemihepatectomy: laparoscopic vs open

**Fig. 2:**
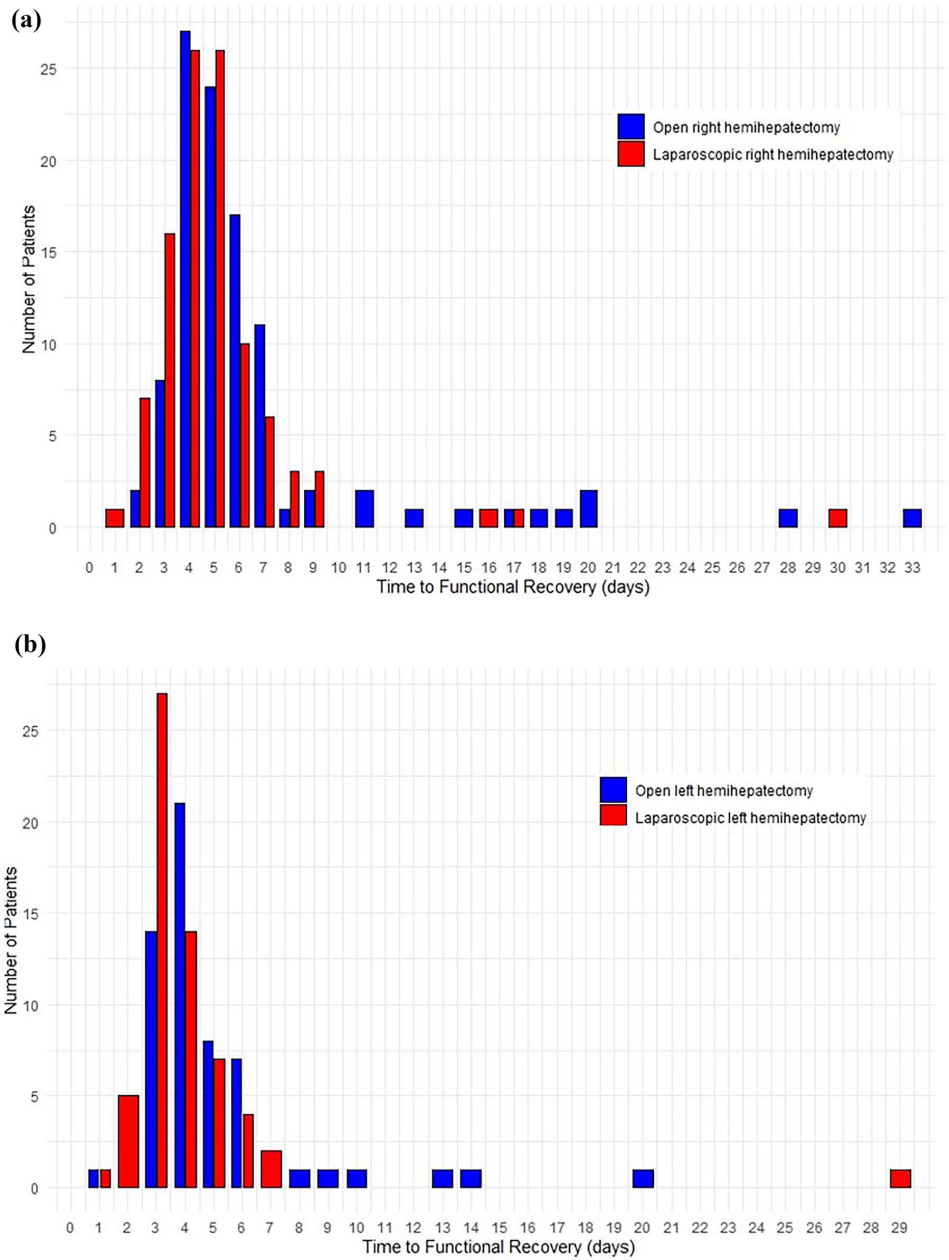
Time to functional recovery in days for Right and left laparoscopic and open hemihepatectomy. **a** Laparoscopic and open Right hemihepatectomy. **b** Laparoscopic and open left hemihepatectomy

Baseline characteristics of the patients undergoing laparoscopic (*n* = 105) and open RH (*n* = 108) were comparable (Table 1). Perioperative outcomes are reported in Table 2. Laparoscopic RH was associated with significantly longer operating time compared to open RH (332 min vs 263 min, *p* < 0.001). However, no significant difference was observed with regard to intraoperative blood loss (500 mL vs 500 mL, *p* = 0.390). Although the difference was statistically significant, the median time to functional recovery was numerically similar between laparoscopic RH (5.0 days, IQR 4.0–5.5) and open RH (5.0 days, IQR 4.0–6.0), (*p* = 0.004), with a mean difference of − 16.8% [95% CI − 27.1 to 4.97%]; *p* = 0.007, favouring laparoscopic RH. Figure 2 shows the distribution for further clarification. Length of hospital stay was 1 day shorter for laparoscopic RH compared to open RH (5.0 days [IQR 4.0–8.0] vs 6.0 days [IQR 5.0–8.0], *p* = 0.014). No significant differences were observed regarding 90-day morbidity, in terms of overall complications, major complications, liver-specific complications or CCI score. The 90-day mortality was similar for both approaches (3.8 vs 3.7%, *p* = 0.304). For patients undergoing resection for a malignancy, there was no difference in the rates of disease-free margin status (R0) between the groups. Overall survival (HR 1.02 [95% CI 0.65 to 1.60], *p* = 0.922) and disease-free survival (HR 0.97 [95% CI 0.67 to 1.42], *p* = 0.893) at a median follow-up of 53 months were not significantly different between both groups.

**Table 1:** Baseline characteristics of laparoscopic vs open right hemihepatectomy

Characteristics	Laparoscopic RH (*n* = 105)	Open RH (*n* = 108)	*p*-value
Sex (male), *n* (%)	58 (55.2)	64 (59.3)	0.553
Age, years, median (IQR)	62 (51–71)	66 (54–72)	0.285
BMI, kgm^−2^, median (IQR)	26 (23–29)	25 (23–28)	0.879
Malignancy, *n* (%)	92 (87.6)	95 (88.8)	0.792
Indication for surgery†, *n* (%)			0.156
Colorectal liver metastasis	65 (61.9)	57 (52.8)	
Hepatocellular carcinoma	9 (8.6)	17 (15.7)	
Cholangiocarcinoma	10 (9.5)	16 (14.8)	
Other malignant	8 (7.6)	5 (4.6)	
Hemangioma	3 (2.9)	6 (5.6)	
Adenoma	3 (2.9)	0 (0.0)	
FNH	1 (1.0)	2 (1.9)	
Other benign	6 (5.7)	5 (4.6)	
ASA Classification, *n* (%)			0.702
I	11 (10.9)	14 (13.2)	
II	59 (58.4)	57 (53.8)	
III	31 (30.7)	34 (32.1)	
IV	0 (0.0)	1 (0.9)	
ECOG performance status score, *n* (%)			0.496
0: asymptomatic, normal activity	76 (73.8)	77 (72.0)	
1: symptomatic, normal activity	24 (23.3)	29 (27.1)	
2: symptomatic, < 50% bedridden	3 (2.9)	1 (0.9)	
3: symptomatic, > 50% bedridden	0 (0.0)	0 (0.0)	
4: 100% bedridden	0 (0.0)	0 (0.0)	
Charlson Comorbidity Index, mean (SD)	7 (5–8)	6 (5–8)	0.271
Previous abdominal surgery, *n* (%)	56 (53.8)	64 (59.8)	0.382
Preoperative portal vein embolization, *n* (%)	15 (14.7)	9 (8.4)	0.154
Preoperative chemotherapy, *n* (%)	39 (38.2)	41 (38.3)	0.990
Additional contralateral surgery, *n* (%)	87 (82.9)	97 (89.8)	0.330
Wedge resection	13 (12.4)	9 (8.3)	
Ablation	3 (2.9)	2 (1.9)	
Ablation and wedge resection	2 (1.9)	0 (0.0)	

†Based on radiological diagnosis

*RH* right hemihepatectomy, *BMI* body mass index, *ASA* American Society of Anesthesiologists, Eastern Cooperative Oncology Group, *FNH* follicular nodular hyperplasia

**Table 2:** Outcomes of laparoscopic vs open right hemihepatectomy

Variables	Laparoscopic RH (*n* = 105)	Open RH (*n* = 108)	ORH vs. LRH^₤^	
*Operative outcomes*				
	*Median (IQR)*		*p-value*^†^	
Operating time, min	332 (279–390)	263 (211–305)	< 0.001*	
Blood loss, mL	500 (300–750)	500 (300–800)	0.390	
	*Number (%)*			
Conversion, *n* (%)	16 (15.2)	–	–	
*Clinical outcomes*				
	*Median (IQR)*		*p-value*^†^	*% difference [95% CI] (p-value)*
Time to functional recovery^¥^, days	5 (4–5.5)	5 (4–6)	0.004*	− 16.8% [− 27.1 to 4.97] (0.007)*
Length of hospital stay^¥^, days	5 (4–8)	6 (5–8)	0.014*	− 13.75% [− 25.1 to 0.70] (0.040)
	*Mean*		*OR [95% CI]*	*p-value*^†^
Complications				
CCI (continuous)	30.0	30.3	0.457 [− 8.804 to 9.717]	0.922
	*Number (%)*			
CCI (> 0)	52 (49.5)	60 (55.6)	0.858 [0.473–1.556]	0.615
Clavien-Dindo > II	16 (15.2)	19 (17.6)	0.972 [0.438–2.157]	0.944
Liver specific morbidity	16 (15.2)	18 (16.7)	1.085 [0.486–2.420]	0.843
30 days readmission	9 (8.6)	10 (9.3)	1.038 [0.377–2.855]	0.943
90 days readmission	15 (14.3)	15 (13.9)	1.249 [0.541–2.886]	0.602
Mortality (within 90 days)	4 (3.8)	4 (3.7)	2.618 [0.418–16.410]	0.304
			*HR [95% CI]*	*p-value*^†^
Overall survival (median FU = 53 months)^**††**^	65 (61.9)	56 (51.9)	1.023 [0.654–1.599]	0.922
Disease-free survival (median FU = 53 months)^**††**^	46 (43.8)	43 (39.8)	0.974 [0.667–1.424]	0.893
	*Number (%)*		*OR [95% CI]*	*p-value*^†^
Disease-recurrence^**††**^				
Recurrence	45 (48.9)	54 (56.8)	0.706 [0.373–1.338]	0.286
Liver-recurrence	25 (27.2)	28 (29.5)	0.756 [0.368–1.554]	0.447
Irradical resection (R1-R2)^**††**^	19 (20.7)	14 (14.7)	1.611 [0.700–3.708]	0.262

*Statistically significant

^**†**^Corrected for confounders: sex, age, centre, and tumour type (benign/malignant)

^**††**^Malignant cases only (ORH *n* = 95; OLH *n* = 50; LRH *n* = 92; LLH *n* = 44)

^¥^Missing for 4 patients in the LRH group

^**₤**^In all analyses either the open or left group are used as the reference group

*CCI* comprehensive complication index, *IQR* interquartile range, *FU* follow-up, *HR* hazard ratio, *LLH* laparoscopic left hemihepatectomy, *LRH* laparoscopic right hemihepatectomy, *OLH* open left hemihepatectomy, *OR* odds ratio, *ORH* open right hemihepatectomy

### Left and right hemihepatectomy

Baseline characteristics and outcomes of patients undergoing RH and LH are presented separately in Supplementary Table 1. Colorectal liver metastasis was the most common surgical indication for both RH (57%)and LH (36%). Portal vein embolization was mostly required in the RH group (12%) and less in the LH group (1%).

No interactions were found between RH and LH, meaning that even though outcomes might differ slightly between the two procedures, the effect of the approach on outcomes were consistent regardless of resection laterality (Table 3).

**Table 3:** Interaction tests of right vs left hemihepatectomy

Outcome	P-interaction for right vs left
	Lap vs open
Time to functional recovery	0.808
Length of hospital stay	0.432
Operating time	0.177
Blood loss*	n.a
Conversion*	n.a
CCI	0.842
Complications	0.837
Liver specific morbidity	0.767
30 day readmission	0.853
90 day mortality	0.884
Overall survival	0.773
Disease-free survival	0.234
Disease recurrence	0.383
Resection margin	0.707

^*^Blood loss and conversion were only analysed using a univariate method, therefore interaction testing was not applicable

To visualize specific outcomes after RH and LH, the descriptive data are shown separately in Supplementary Table 2. A clinically relevant difference in operating time was noted. RH takes median 23 min longer when performed open (263 min and 240 min) and median 47 min longer when performed laparoscopically (332 min and 275 min). Across both surgical approaches median time to functional recovery and hospital stay was one day longer. The rate of complications, mortality and long-term survival were almost identical for RH and LH.

Conversion rate for laparoscopic RH and LH were 15% and 20%, respectively. The reasons for conversion are displayed in Supplementary Table 3. Liver specific morbidity per hemihepatectomy side and surgical approach is displayed in Supplementary Table 4a and 4b. Noticeably, there is a higher occurrence of ascites (*n* = 12 vs *n* = 1), liver failure (*n* = 10 vs *n* = 1) and pleural effusion (*n* = 20 vs *n* = 1) in RH.

## Discussion

This post hoc subgroup analysis of right hemihepatectomy in the ORANGE II PLUS multicentre randomized controlled trial, found modest, population-level benefits of laparoscopy compared to open surgery, including distribution-based quicker time to functional recovery and a median one day shorter length of hospital stay. Differences in time to functional recovery were small, and should not be expected on individual patient-level. Moreover, laparoscopy showed significantly longer operating time. No differences were observed in other perioperative or oncological outcomes. Interaction testing between surgical approach and hemihepatectomy laterality did not reach significance, suggesting that the impact of approach on outcomes was consistent for both RH and LH.

The observed benefits of laparoscopy for RH are consistent with outcomes observed across other liver resection procedure types [5–8]. This suggests that the advantages of laparoscopic surgery are preserved despite the increased complexity of RH. However, the substantial physiological impact of RH may attenuate the positive effects of smaller incisions, as reflected by only marginal improvements in time to functional recovery. These modest improvements ultimately did not translate into a full day’s reduction in median recovery time, as previously observed in the results of the ORANGE II PLUS trial [5]. Although the median recovery time was numerically identical (5 vs. 5 days), the statistical difference likely reflects variation in distribution. Even if modest, such differences may still be clinically and systemically relevant when scaled across larger patient populations, but is not likely to show individual patient-level differences. The similarities in the postoperative outcomes of laparoscopic and open RH demonstrate no significantly larger risks of the laparoscopic approach for RH, thus supporting the safety and feasibility of its introduction in experienced hands. Although a meta-analysis of retrospective studies previously reported lower complication rates and less severe morbidity for laparoscopic compared to open RH these findings were not replicated in the present randomized study [15].

The present study found longer operating time for laparoscopic RH as compared to the open approach, which is consistent with findings from other randomized trials comparing laparoscopy with open liver surgery [5, 6, 8]. Prolonged operative time may carry implications for operative scheduling and procedural costs. Despite longer operating times, cost-effectiveness analyses from the ORANGE II PLUS and Oslo COMET trials demonstrated that laparoscopic liver resection still has a high probability of being cost-effective, owing to benefits such as shorter hospital stays and improvements in quality of life [6, 29]. However, given that the improvements in outcomes, such as time to functional recovery, are less pronounced within the RH subgroup, and that differences in operating time are relatively larger, cost-effectiveness conclusions may be less apparent for this group.

The higher technical complexity and larger impact on the patient of RH as opposed to patients who require a LH are visualized in clinically relevant longer time to functional recovery, length of hospital stay, and operating time across both laparoscopic and open approaches. Other postoperative or oncological outcomes yielded no such differences. Therefore, this subgroup analysis, indicates that, although RH is a more technically demanding procedure, laparoscopy does not seem to add additional risk when performed by experienced surgeons in comparison to when LH is performed. A prolonged length of stay was also noted for patients undergoing RH compared to LH for cancer in a study by Douaiher et al. [17] In the present study, notably, more complications such as ascites, liver failure and pleural effusion are seen in RH. These complications are thought to occur more frequently after RH due to the typically larger resected liver volume leaving smaller future liver remnants, and the closer proximity, and greater manipulation of, the diaphragm. Nobili et al. also found higher rates of pleural effusion following resections of the right liver lobe, and observed higher rates of complications across both laparoscopic and open procedures [30]. The current study had an overall 90-day mortality rate of 3%, which is consistent with rates observed in the current literature on major liver resections [1, 21, 22, 31, 32]. Mortality rates were very similar for RH and LH. Conversely, Douaiher et al. and Karanjia et al. both found higher mortality for right-sided compared to left-sided resection. However, Karanjia et al. did compare (extended) RH to all other liver resections, including smaller left-sided resections such as segmentectomies and wedge resections [22].

Laparoscopy has been proposed as the gold standard for left lateral sectionectomy and anterior segment resections, however recommendations for the use of laparoscopy in RH are more cautious [13, 33–36]. These results suggest that when adopted by surgeons in high-volume centres with sufficient experience, laparoscopic RH can result in improved patient outcomes compared to open RH. This study was performed in high-volume experienced centres across Europe, where each participating surgeon had extensive experience in laparoscopic minor liver resections and performed at least ten laparoscopic hemihepatectomies prior to trial initiation, the results of this study do not justify universal adoption, but rather support its benefits in experienced settings. Extrapolation of these findings beyond this setting should therefore be undertaken with caution and under meticulous proctoring. As centres progress in their learning curve it can be expected that certain disadvantages of laparoscopic RH such as longer operating time will lessen [37, 38].

Certainly, this study is not without limitations. First, this is a *post-hoc* analysis of a randomized study which may introduce inherent biases. Second, although the trial was stratified for hemihepatectomy side, comparisons between RH and LH are not randomized and are hence subject to selection bias. Specifically, outcomes of RH may have been influenced by a higher proportion of malignancy, more preoperative portal vein embolization, and other potential unmeasured confounders. Third, patients were excluded when laparoscopic hepatectomy was deemed technically unfeasible by a multidisciplinary team, limiting the generalizability of these findings. Fourth, as a subgroup analysis, the study is underpowered, with a smaller sample size than the original trial, therefore results are mostly descriptive and do not provide definitive conclusions, and should thus be interpreted with caution. Fifth, although robot-assisted liver surgery is rapidly expanding within the field of minimally invasive hepatectomy, it remains uncertain to what extent the findings of this study can be directly extrapolated to robotic procedures, and therefore, warrants further research.

In conclusion, the results of this study suggest that, when patients are appropriately selected and the procedure is performed by adequately trained surgeons, laparoscopic RH is associated with favourable short-term clinical outcomes without compromising long-term oncological results as compared to open RH. However, gains are small and most likely population-based rather than on an individual patient-level, and the technical complexity of laparoscopic RH should not be underestimated. Emphasis must be placed on appropriate training, sufficient procedural volume and specialized expertise in centres undertaking these high-complexity operations.

## Supplementary Information

Below is the link to the electronic supplementary material.

Supplementary file1 (PDF 2249 KB)

Supplementary file2 (PDF 1864 KB)

Supplementary file3 (DOCX 28 KB)

## Data Availability

Data collected for the study, including de-identified individual participant data and a data dictionary defining each field in the set, can be made available to others on reasonable request and after signing appropriate data sharing agreements after all following studies on this main paper by the research team have been concluded. Please send data access requests to r.van.dam@mumc.nl. Such requests must be approved by the respective ethics boards and appropriate data custodians.
