# Supplementary material for: Laparoscopic versus open hemihepatectomy: does side matter? A post-hoc analysis of the ORANGE II PLUS randomized controlled trial
Source: Surg Endosc. 2026 Mar 9;40(5):4195–205. doi: 10.1007/s00464-026-12588-w (PMC13161259; doi:10.1007/s00464-026-12588-w)
Supplement: Supplementary file 3 — Supplementary file3 (DOCX 28 KB) [file 464_2026_12588_MOESM3_ESM.docx]

# Appendix

Supplementary Table 1: Baseline characteristics of right and left hemihepatectomy

| Characteristics | RH (n=213) | LH (n=119) |
| --- | --- | --- |
| Sex (male), n (%) | 122 (57.3) | 73 (61.3) |
| Age, years, median (IQR) | 64 (52 – 72) | 64 (54 – 71) |
| BMI, kgm^-2^, median (IQR) | 25 (23 – 29) | 25 (23 – 28) |
| Malignancy, n (%) | 187 (88.2) | 94 (79.0) |
| Indication for surgery†, n (%)  Colorectal liver metastasis  Hepatocellular carcinoma  Cholangiocarcinoma  Other malignant  Hemangioma  Adenoma  FNH  Other benign | 122 (57.3)  26 (12.2)  26 (12.2)  9 (4.2)  3 (1.4)  3 (1.4) | 43 (36.1)  21 (17.6)  21 (17.6)  4 (3.4)  3 (2.5)  0 (0.0) |
| ASA-classification, n (%)  I  II  III  IV | 25 (12.1)  116 (56.0)  65 (31.4)  1 (0.5) | 7 (6.1)  69 (60.0)  39 (33.9)  0 (0.0) |
| ECOG performance status score, n (%)  0: asymptomatic, normal activity  1: symptomatic, normal activity  2: symptomatic, <50% bedridden  3: symptomatic, >50% bedridden  4: 100% bedridden | 153 (72.9)  53 (25.2)  4 (1.9)  0 (0.0) | 91 (78.4)  23 (19.8)  1 (0.9)  1 (0.9) |
| Charlson comorbidity index, median (IQR) | 5 (7 – 8) | 6 (4 – 8) |
| Previous abdominal surgery, n (%) | 120 (56.9) | 59 (49.6) |
| Preoperative portal vein embolization, n (%) | 24 (11.5) | 1 (0.8) |
| Preoperative chemotherapy, n (%) | 80 (38.3) | 37 (31.1) |
| Additional contralateral surgery, n (%)  Wedge resection  Ablation  Ablation and wedge resection | 24 (11.2)  5 (2.3)  2 (0.9) | 12 (10.1)  4 (3.4)  2 (1.7) |
| Approach, n (%)  Open  Laparoscopic | 108 (50.7)  105 (49.3) | 58 (48.7)  61 (51.3) |
| ASA, American Society of Anaesthesiologists; BMI, body mass index; ECOG, Eastern Cooperative Oncology Group; LH, left hemihepatectomy; RH, right hemihepatectomy  *Statistically significant, †Based on radiological diagnosis | | |

Supplementary Table 2 Outcomes of right versus left hemihepatectomy

| Variables | ORH  (n=108) | OLH  (n=58) | LRH  (n=105) | LLH  (n=61) |
| --- | --- | --- | --- | --- |
| Operative outcomes | | | | |
|  |  | | | |
| Operating time, min | 263  (211-305) | 240  (168-275) | 332  (279-390) | 275  (230-338) |
| Blood loss, mL | 500  (300-800) | 325  (200-600) | 500  (300-750) | 400  (200-850) |
|  |  | | | |
| Conversion, n (%) | - | - | 16 (15.2) | 12 (19.7) |
| Clinical outcomes | | | | |
|  | **Median (IQR)** | | | |
| Time to functional recovery^¥^, days | 5 (4-6) | 4 (3-5) | 5 (4-5.5) | 3 (3-4) |
| Length of hospital stay^¥^, days | 6 (5-8) | 5 (4-6) | 5 (4-8) | 4 (4-6) |
|  | **Mean** | | | |
| Complications  CCI (continuous) | 30.3 | 29.8 | 30.0 | 28.4 |
|  | **Number (%)** | | | |
| CCI (>0) | 60 (55.6) | 20 (34.5) | 52 (49.5) | 22 (36.1) |
| Clavien-Dindo >II | 19 (17.6) | 9 (15.5) | 16 (15.2) | 8 (13.1) |
| Liver specific morbidity | 18 (16.7) | 8 (13.8) | 16 (15.2) | 7 (11.5) |
| 30 days Readmission | 10 (9.3) | 2 (3.4) | 9 (8.6) | 4 (6.6) |
| 90 days Readmission | 15 (13.9) | 5 (8.6) | 15 (14.3) | 7 (11.5) |
| Mortality (within 90 days) | 4 (3.7) | 1 (1.7) | 4 (3.8) | 1 (1.6) |
|  |  | | | |
| Overall Survival (median FU=53 months)^††^ | 56 (51.9) | 32 (55.2) | 65 (61.9) | 38 (62.3) |
| Disease-free Survival (median FU=53 months)^††^ | 43 (39.8) | 21 (36.2) | 46 (43.8) | 30 (49.2) |
|  | **Number (%)** | | | |
| Disease-recurrence^††^  Recurrence  Liver-recurrence | 54 (56.8)  28 (29.5) | 30 (60.0)  22 (44.0) | 45 (48.9)  25 (27.2) | 21 (47.7)  11 (25.0) |
| Irradical resection (R1-R2) ^††^ | 14 (14.7) | 5 (10.0) | 19 (20.7) | 8 (18.2) |

Supplementary Table 3: Reasons for conversion to open surgery.

| **Conversions reason** | RH  (n=16) | LH  (n=12) |
| --- | --- | --- |
| Urgent |  |  |
| Bleeding | 4 | 2 |
| Splenic Rupture | 0 | 1 |
| Non-urgent |  |  |
| Oncological uncertainty | 6 | 5 |
| Unclear anatomy | 1 | 1 |
| Adhesions | 2 | 1 |
| Inadequate dimensions | 1 | 1 |
| Technical problems | 2 | 0 |
| Portal thrombectomy needed | 0 | 1 |

Supplementary Table 4a: Complications; liver specific.

| **Composite endpoint**  **Liver specific complications** | ORH  (n=108) | OLH  (n=58) | LRH  (n=105) | LLH  (n=61) |
| --- | --- | --- | --- | --- |
| Post-hepatectomy liver failure | 5 | 0 | 5 | 1 |
| Ascites | 6 | 1 | 6 | 0 |
| Intra-abdominal abscess | 3 | 3 | 4 | 2 |
| Bile leakage | 3 | 4 | 1 | 3 |
| Intra-abdominal haemorrhage | 1 | 0 | 1 | 1 |
| Operative mortality | 0 | 0 | 0 | 0 |

Supplementary Table 4b: Complications; other.

| **Other intervention related complications** | ORH  (n=108) | OLH  (n=58) | LRH  (n=105) | LLH  (n=61) |
| --- | --- | --- | --- | --- |
| Sepsis | 5 | 3 | 2 | 2 |
| Pulmonary embolus | 0 | 0 | 2 | 0 |
| Pleural Effusion | 11 | 0 | 9 | 1 |
| Gastroparesis | 1 | 2 | 0 | 0 |
| Post-operative ileus | 1 | 0 | 2 | 0 |
| Cardiac arrest | 0 | 1 | 0 | 0 |

Supplementary Table 5: ORANGE II PLUS collaborative

| **ORANGE II PLUS collaborative** | | |
| --- | --- | --- |
| **Centre** | **Name** | **Role** |
| Aachen University Hospital  Aachen, Germany | Ulf Neumann | Principal investigator |
|  | Florian Ulmer | Medical staff involved in patient care |
|  | Finja Clausen | Research nurse |
| Aintree University Hospital NHS Foundation Trust  Aintree, United Kingdom | Rafael Díaz-Nieto | Principal investigator |
|  | Michelle Lintforth | Research nurse |
| Amsterdam University Medical Centres  Amsterdam, The Netherlands | Marc Besselink | Principal investigator |
|  | Pieter Tanis | Medical staff involved in patient care |
|  | Burak Gorçek | PhD candidate |
|  | Marcel van der Poel | PhD candidate |
|  | Gabriela Pilz da Cunha | PhD candidate |
| University Hospitals Birmingham NHS Trust  Birmingham, United Kingdom | Robert Sutcliffe | Principal investigator |
|  | Ravi Marudanayagam | Medical staff involved in patient care |
|  | Penelope Rogers | Research nurse |
| Erasmus Hospital, Brussels, Belgium | Valerio Lucidi | Principal investigator |
|  | Viviane van Laethem | Research nurse |
| Ghent University Hospital  Ghent, Belgium | Roberto Troisi | Principal investigator |
|  | Frederik Berrevoet | Medical staff involved in patient care |
|  | Vincenzo Scuderi | Medical staff involved in patient care |
|  | Aude Vanlander | Medical staff involved in patient care |
|  | Betsy van Loo | Research nurse, trial coordinator |
|  | Kathleen Segers | Research nurse |
| Jessa Hospital  Hasselt, Belgium | Gregory Sergeant | Principal investigator |
| Groeninge General Hospital Kortrijk, Belgium | Mathieu D’Hondt | Principal investigator |
|  | Celine Demeyere | Research nurse |
| King’s College Hospital NHS Foundation Trust  London, United Kingdom | Krishna Menon | Principal investigator |
|  | Ane Zamalloa | Research nurse |
| Maastricht University Medical Centre+  Maastricht, The Netherlands | Ronald van Dam | Principal investigator, trial coordinator |
|  | Cornelis Dejong | Medical staff involved in patient care |
|  | Lloyd Brandts | Trial statistician |
|  | Robert Fichtinger | PhD candidate, trial coordinator |
|  | Bram Olij | PhD candidate |
|  | Merel Kimman | Health economics and Quality of life expert |
|  | Remon Korenblik | PhD candidate |
| Maastricht University  Maastricht, The Netherlands | Gerard van Breukelen | Trial statistician |
| San Raffaele Hospital Milan, Italy | Luca Aldrighetti | Principal investigator |
|  | Francesca Ratti | Medical staff involved in patient care |
| Newcastle upon Tyne Hospitals NHS Foundation Trust  Newcastle, United Kingdom | Steve White | Principal investigator |
|  | Stuart Robinson | Medical staff involved in patient care |
|  | Caroline Brunton | Research nurse |
| Oslo University Hospital  Oslo, Norway | Björn Edwin | Principal investigator |
|  | Åsmund Fretland | Medical staff involved in patient care |
|  | Davit Aghayan | PhD candidate |
| Oxford University Hospitals NHS Foundation Trust  Oxford, United Kingdom | Zahir Soonawalla | Principal investigator |
|  | Katherine Gordon-Quayle | Research nurse |
| University Hospitals Plymouth NHS Foundation Trust  Plymouth, United Kingdom | Somaiah Aroori | Principal investigator |
|  | Tracy Ward | Research nurse |
| University Hospital Southampton NHS Foundation Trust  Southampton, United Kingdom | John Primrose | Principal investigator |
|  | Mohammad Abu Hilal | Principal investigator |
|  | Christopher Kuemmerli | PhD candidate |
|  | Jess Boxal | Research nurse |
| Southampton Clinical Trials Unit  Southampton, United Kingdom | Zina Eminton | Trial coordinator |
